# Quantitative Proteomic and Microcystin Production Response of *Microcystis aeruginosa* to Phosphorus Depletion

**DOI:** 10.3390/microorganisms9061183

**Published:** 2021-05-31

**Authors:** Nian Wei, Lirong Song, Nanqin Gan

**Affiliations:** 1 State Key Laboratory of Freshwater Ecology and Biotechnology, Institute of Hydrobiology, Chinese Academy of Sciences, Wuhan 430072, China; weinian710@163.com; 2 Yangtze River Fisheries Research Institute, Chinese Academy of Fishery Sciences, Wuhan 430072, China

**Keywords:** cyanobacterial bloom, *Microcystis aeruginosa*, phosphorus depletion, proteomics, microcystin

## Abstract

*Microcystis* blooms are the most widely distributed and frequently occurring cyanobacterial blooms in freshwater. Reducing phosphorus is suggested to be effective in mitigating cyanobacterial blooms, while the underlying molecular mechanisms are yet to be elucidated. In the present study, isobaric tags for relative and absolute quantitation (iTRAQ)-based quantitative proteomics was employed to study the effects of phosphorus depletion on *Microcystis aeruginosa* FACHB-905. The production of microcystins (MCs), a severe hazard of *Microcystis* blooms, was also analyzed. In total, 230 proteins were found to be differentially abundant, with 136 downregulated proteins. The results revealed that, upon phosphorus limitation stress, *Microcystis aeruginosa* FACHB-905 raised the availability of phosphorus primarily by upregulating the expression of orthophosphate transport system proteins, with no alkaline phosphatase producing ability. Phosphorus depletion remarkably inhibited cell growth and the primary metabolic processes of *Microcystis*, including transcription, translation and photosynthesis, with structures of photosystems remaining intact. Moreover, expression of nitrogen assimilation proteins was downregulated, while proteins involved in carbon catabolism were significantly upregulated, which was considered beneficial for the intracellular balance among carbon, nitrogen and phosphorus. The expression of MC synthetase was not significantly different upon phosphorus depletion, while MC content was significantly suppressed. It is assumed that phosphorus depletion indirectly regulates the production of MC by the inhibition of metabolic processes and energy production. These results contribute to further understanding of the influence mechanisms of phosphorus depletion on both biological processes and MC production in *Microcystis* cells.

## 1. Introduction

Cyanobacterial blooms have long been an aquatic environmental problem of many freshwater bodies over wide geographical areas, especially in China, with *Microcystis* as the most common genera [1]. Nitrogen and phosphorus are involved in the proliferation of phytoplankton as high nitrogen and phosphorus concentrations commonly lead to the occurrence of blooms [2]. In particular, the occurrence of cyanobacterial blooms in eutrophic lakes frequently coincides with an increase in phosphorus in the water column [3]. In addition, phosphorus was considered the first limiting nutrient element of algal populations and concentrations in lakes [4,5]. Much research has discovered that reducing phosphorus was effective in reducing eutrophication and cyanobacterial blooms [6,7,8].

Phosphorus is a fundamental element incorporated into the primary structural and functional macromolecules in cyanobacterial cells. It is a major element in nucleic acids, acts as a universal energy carrier in the form of adenosine triphosphate (ATP) and nicotinamide adenine dinucleotide phosphate (NADP), and plays a role in signaling communication systems involving phosphorylation. The influence of phosphorus limitation or depletion on *Microcystis* cells have been investigated in several studies. Under phosphorus limitation conditions, the cellular protein content was lower and the photosynthetic efficiency was distinctly reduced [9,10], which was the same as the growth rate and chlorophyll a content. Additionally, more alkaline phosphatase (ALP) was secreted to obtain sufficient phosphorus for growth [10,11]. Several studies also discovered that the length/diameter and volume of *Microcystis* cells increased at lower phosphorus concentrations, with higher carbohydrate content per cell [12,13]. Most of these results were biochemical or physiological responses upon phosphorus depletion, while the underlying molecular mechanisms were still not clear. To date, few investigations have focused on the cellular-wide response of *Microcystis* cells to phosphorus limitation. Therefore, a systematic and comprehensive investigation at the molecular level would assist in understanding primary changes in bloom-forming *Microcystis* cells under phosphorus limitation conditions.

Proteins are directly in charge of cellular metabolism and growth. Thus, the analysis of changes at the protein level is generally considered to provide crucial information for the understanding of cellular metabolic pathways. At the protein level, changes in regulators, their regulation products and the protein-protein interaction network could be discovered at the same time. Proteomic research focuses on the expression of the whole proteome, which is a powerful approach to investigate stress responses. Proteomic investigation has been used in several cyanobacteria species to study their phosphorus limitation or starvation responses, including *Anabaena* [14,15], *Synechocystis* [16] and *Trichodesmium erythraeum* [17]. Broad proteomic changes in *Microcystis* cells under phosphorus limitation conditions have been investigated only by two-dimensional electrophoresis (2-DE) method, with limited detectability and low sensitivity [18], which discovered that upon phosphorus starvation, proteins involved in protein synthesis and carbon assimilation were downregulated, while those related to photoprotection were upregulated. A high-throughput quantitative proteomic method would provide more information in understanding the underlying mechanism of the phosphorus limitation response in *Microcystis* cells. Isobaric tags for relative and absolute quantitation (iTRAQ) is a mature, effective and extensively used method for high-throughput protein quantification [19], with significant advantages of high reproducibility and sensitivity [20]. Its application to the investigation into the proteomic responses of *Microcystis* has been demonstrated reliable and informative [21,22].

Hepatotoxic microcystins (MCs), primarily produced by *Microcystis*, have been a serious worldwide hazard in lakes that suffer from high-frequency and long-term *Microcystis* blooms [1]. Field research around the world has discovered that phosphorus enrichment promoted both *Microcysits* biomass and MCs production [23,24,25]. Furthermore, low level of phosphorus was often correlated with less MCs [23,24,26]. Several experiments have been conducted to verify the relationship between phosphorus limitation and MCs production and explore the underlying mechanisms. Most of the investigations have identified that phosphorus depletion has a relevant impact on MCs production, though with conflicting results. Phosphorus limitation might reduce or induce the production of MCs, under various limitation conditions with different species used [27,28]. Furthermore, it was supposed that the influence of phosphorus limitation on cell growth rate, the *mcy* gene transcription, the MC-producing rate and the content of MCs passed to daughter cells during cell division of *Microcystis* cells might be the possible reasons [12,27,29,30], while the mechanisms on the protein level were still lacking. Therefore, we supposed that combining the proteome results with intracellular and extracellular MC content might further elucidate how phosphorus limitation affects the production of MCs.

In the present study, we employed the iTRAQ-based quantitative proteomic method to study the effects of phosphorus limitation on *Microcystis* cells. The intracellular and extracellular MC content were also analyzed. This investigation contributes to the understanding of global cellular responses of *Microcystis* to phosphorus limitation.

## 2. Materials and Methods

### 2.1. Cultures and Sampling

*Microcystis aeruginosa* FACHB-905 was provided by the Freshwater Algae Culture Collection of the Institute of Hydrobiology (FACHB-collection, Wuhan, China). The cultures were grown in BG-11 medium at 25 °C and the light intensity was set at 25 µmol photons m^−2^ s^−1^ with a 12 h: 12 h light: dark cycle. MC variants of strain FACHB-905 were removed from culture and sampled to the high-performance liquid chromatography (HPLC) methodology, and the result discovered that MC-LR was the only MC isoform.

Cells in the exponential growth phase were collected and washed three times in phosphorus-free BG-11_0_ medium (BG-11 medium without K_2_HPO_4_; potassium of K_2_HPO_4_ was supplied by equimolar KCl). The washed cells were cultivated in BG-11_0_ medium for 7 days to exhaust the stored phosphorus. After 7 days of phosphorus starvation, cells were harvested by centrifugation at 3000 g for 5 min and seeded in BG-11 medium (300 mL cultures in 500 mL Erlenmeyer flasks) at different phosphorus levels, which were set to 5.4 mg L^−1^ (phosphorus level of the BG-11 medium), 0.54 mg L^−1^, 0.054 mg L^−1^ and 0 mg L^−1^ in the form of K_2_HPO_4_. Potassium was supplied with equimolar KCl to maintain the ion concentration. Cultures were sampled on days 1, 3, 5 and 7. Aliquots of 20–50 mL were conducted for two centrifugation runs at 3000 g for 6 min. The obtained cell pellets were pooled and washed once with sterile distilled water, followed by liquid nitrogen refrigeration and lyophilization. Samples were stored at −80 °C until intracellular MC extraction. Meanwhile, the supernatants from the two centrifugation runs were combined and filtered through a GF/C glass microfiber membrane with 1.2 μm particle retention (Whatman, GE Healthcare, Chicago, IL, USA). The filtrate was instantly processed for extracellular MC concentrating.

### 2.2. Cell Counting

On the day of sampling, aliquots of 1 mL were fixed with 1 μL Lugol’s solution. Cells in the fixed samples were counted under an Eclipse E200 light microscope (Nikon, Tokyo, Japan) with a hemocytometer (QiuJing, Shanghai, China). For each sample, the counting was performed six times with a maximum deviation of approximately 20%, and the cell concentration was reported as the average cell number.

### 2.3. Chlorophyll a and Carotenoid Analysis

On the seventh day, 5–20 mL cultures were filtered onto a GF/C glass microfiber membrane (Whatman) and extracted with 5 mL 80% (*v*/*v*) acetone in dark at 4 °C for 24 h. The concentrations of chlorophyll a and carotenoids were determined spectrophotometry [31].

### 2.4. MC Analysis

Extracellular MC concentrating, intracellular MC extraction and MC content determination were processed as described in details in our previous study [32]. Briefly, the extracellular MC in the filtrate was cleaned and concentrated by solid phase extraction on a C18 Sep-Pak cartridge (Waters, Milford, MA, USA), and the intracellular MCs were extracted once from lyophilized cells with 5% acetic acid (*v*/*v*) and twice with 85% methanol (*v*/*v*). The MC concentration in the samples were analyzed by HPLC on an Alliance Waters™ e2695 (Waters, Milford, MA, USA) separation module coupled with a photodiode array (PDA) detector using a C18 cartridge (XBridge C18 Column, 4.6 × 250 mm, 5 μm, Waters, Milford, MA, USA). MCs were identified based on characteristic UV absorption at 238 nm by monitoring UV spectra from 200 to 400 nm, and further quantified according to a standard curve established from linear regression values of MC-LR standard (Sigma-Aldrich, St. Louis, MO, USA).

### 2.5. Protein Preparation, Digesting and iTRAQ Labeling

Cultures of the 5.4 mg L^−1^, 0.054 mg L^−1^ and 0 mg L^−1^ groups growing for 7 days were chosen for iTRAQ analysis. Two biological replicates from the 5.4 mg L^−1^ group and three biological replicates from each 0.054 mg L^−1^ and 0 mg L^−1^ group were combined into an 8-plex iTRAQ experiment.

The cells were collected by centrifugation (3000 g, 4 °C, 5 min), washed twice with precooled sterile MilliQ water and immediately frozen in liquid nitrogen. Frozen samples were ground to powder in liquid nitrogen, suspended in five-time precooled TCA/acetone (*v:v* = 1:9) and kept at −20 °C for 4 h, followed by centrifugation at 6000 g for 40 min. The pellet was washed three times with cold acetone and then air dried at 4°C. The precipitated proteins were dissolved in 300 µL of SDT lysis buffer (4% (*w*/*v*) SDS, 1 mmol/L dithiothreitol (DTT), 100 mmol/L Tris-HCl, pH 7.6). Protein concentration was determined by a bicinchoninic acid (BCA) protein assay kit (Beyotime, Shanghai, China). Protein digestion was performed according to the filter-aided sample preparation (FASP) method [33]. The obtained peptide mixtures (100 µg) of each sample were then labeled with the 8-plex iTRAQ reagent following the manufacturer’s instructions (Applied Biosystems, Foster City, CA, USA).

### 2.6. Sample Fractionation and Liquid Chromatography-Tandem Mass Spectrometry (LC-MS/MS) Analysis

Labeled peptides were mixed into one sample mixture and fractionated by strong cation-exchange (SCX) chromatography in the AKTA Purifier System (GE Healthcare, Chicago, IL, USA). The peptide mixture was vacuum dried, acidified with buffer A (10 mmol/L KH_2_PO_4_ in 25% (*v*/*v*) ACN, pH 3.0) and fractioned by a Polysulfoethyl™ (PolyLC Inc., Columbia, MD, USA) column (4.6 × 100 mm, 5 µm, 200 Å). A gradient elution (0–22 min buffer B (500 mmol/L KCl, 10 mmol/L KH_2_PO_4_ in 25% ACN, pH 3.0)) 0–8%; 22–47 min buffer B 8–52%; 47–50 min buffer B 52–100%; 50–58 min buffer B 100%; after 58 min buffer B 0%) at a flow rate of 1 mL min^−1^ was used to separate the peptides. The chromatograms were monitored at 214 nm. Eluent was collected every minute and integrated into 15 fractions. Each fraction was desalted, vacuum dried and reconstituted in 0.1% (*v*/*v*) formic acid (FA).

LC-MS/MS analysis were performed by an Easy nLC system (Proxeon Biosystems, Thermo Scientific, Waltham, MA, USA) coupled to a Q Exactive MS (Thermo Scientific). The peptide mixture in each fraction was loaded onto a reversed-phase trap column (Thermo Scientific Acclaim PepMap100, 100 µm × 2 cm, nanoViper C18, Waltham, MA, USA), and separated by a C18 reversed-phase analytical column (Thermo Scientific Easy Column, 75 µm × 10 cm, 3 µm resin, Waltham, MA, USA) with a 60 min linear gradient at a flow rate of 300 nL min^−1^. The mobile phase consisted of buffer A (0.1% FA) and buffer B (0.1% FA in 84% ACN). The linear gradient was performed as follows: 0%–35% buffer B for 50 min, 35–100% buffer B for 5 min and finally 100% buffer B for 5 min.

Mass spectra were acquired over the mass/charge ratio (*m*/*z*) range of 300–1800 at a resolution of 70,000 at *m*/*z* 200 in positive ion mode with the automatic gain control target value set to 3 × 10^6^ and the dynamic exclusion duration set to 40.0 s. From the survey scan, the top 10 most abundant precursor ions were selected for higher energy collisional dissociation (HCD) fragmentation at a resolution of 17,500 at *m*/*z* 200, with the normalized collision energy set at 30 eV and the underfill ratio defined as 0.1%.

### 2.7. Protein Identification and Quantitation

MS/MS spectra data were searched using MASCOT software (version 2.3.02, Matrix Science, London, UK) and Proteome Discoverer 1.4 (Thermo Scientific) against the UniProt database (uniprot-microcystis_aeruginosa_dianchi905.fasta, 5445 sequences). All data used for protein identification were evaluated by the false discovery rate (FDR, ≤1%) analysis. The reporter ion-based quantitation (RIQ) method was employed for iTRAQ quantitation, namely, the weighted average of the intensities of reporter ions in each identified peptide was used as the reference. The protein ratios were calculated as the median of only the unique peptides. Only proteins detected in all runs were included in the protein quantitation dataset. Statistical significance was measured using the Student’s *t* test, with an FDR correction (set as 5%), and differences were considered significant if the *p*-value was less than 0.05. The fold change (iTRAQ ratio) was calculated as the average of three biological replicates. Proteins with more than a 1.5-fold change containing at least two unique peptides and a *p*-value below 0.05 were recognized as significantly differentially expressed proteins. The mass spectrometry proteomics data have been deposited to the ProteomeXchange Consortium via the PRIDE [34] partner repository with the dataset identifier PXD025188.

### 2.8. Bioinformatics Analysis

Sequence information of the identified proteins was retrieved from the UniProt database (http://www.uniprot.org/ (accessed on 29 August 2016)). The retrieved sequences were locally searched against the Swiss-Prot database (nonredundant (nr) database) by NCBI BLAST+ client software (ncbi-blast-2.2.28 +-win32.exe). In the present study, the top 10 blast hits with E-values less than 1 × 10^−3^ for each query sequence were retrieved and subjected to Gene Ontology (GO) mapping and annotation by Blast2GO (Version 3.0.1). The sequences without BLAST hits and those unannotated sequences were further analyzed by InterProScan against the EBI databases for functional annotation. Annotation augmentation was also performed by ANNEX, which established connections among different GO terms to increase annotation accuracy. Metabolic pathway data of identified proteins were obtained from the Kyoto Encyclopedia of Genes and Genomes (KEGG, http://www.genome.jp/kegg/ (accessed on 8 September 2016)) database.

## 3. Results

### 3.1. Microcystis Growth

**Figure 1:**
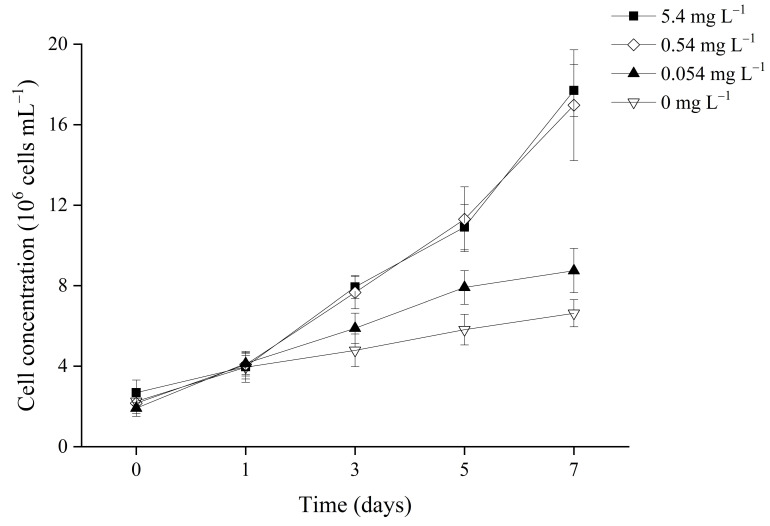
Changes in cell concentration in cultures of *Microcystis aeruginosa* FACHB-905 at different initial phosphorus concentrations. Lines with different symbols represent different initial phosphorus concentrations. Error bars indicate standard deviation from three biological replicates.

Before treatment with different phosphorus concentrations, *Microcystis* cells underwent phosphorus starvation treatment for 7 days. This treatment exhausted stored phosphorus inside cells, including the polyphosphate body, synchronizing cells to the same intracellular phosphorus level [35]. The growth of cells incubated at different initial phosphorus concentrations exhibited relative differences. As shown in Figure 1, after one day, the cell concentration of all cultures rose to the same level, approximately 4 × 10^6^ cells mL^−1^. Thereafter, cells in the 5.4 mg L^−1^ and 0.54 mg L^−1^ groups grew exponentially, while those in the 0.054 mg L^−1^ and 0 mg L^−1^ groups grew linearly. On the seventh day, the cell concentrations in the 5.4 mg L^−1^ and 0.54 mg L^−1^ cultures were twice/triple those of the 0.054 mg L^−1^ and 0 mg L^−1^ cultures.

### 3.2. Pigment Content

**Figure 2:**
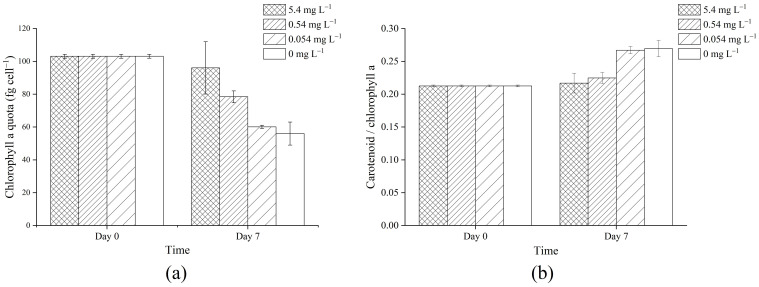
Changes in chlorophyll a content (**a**) and carotenoid/chlorophyll a ratio (**b**) in cultures of *Microcystis aeruginosa* FACHB-905 after 7 days of growth at different initial phosphorus concentrations. Columns with different filling represent different initial phosphorus concentrations. Error bars indicate standard deviation from three biological replicates.

After 7 days of growth, the chlorophyll a content per cell decreased with less available phosphorus in the medium (Figure 2a). The carotenoid/chlorophyll ratio was significantly higher in the 0.054 mg L^−1^ and 0 mg L^−1^ treatments than in the 5.4 mg L^−1^ and 0.54 mg L^−1^ treatments (Figure 2b).

### 3.3. MC Concentrations

**Figure 3:**
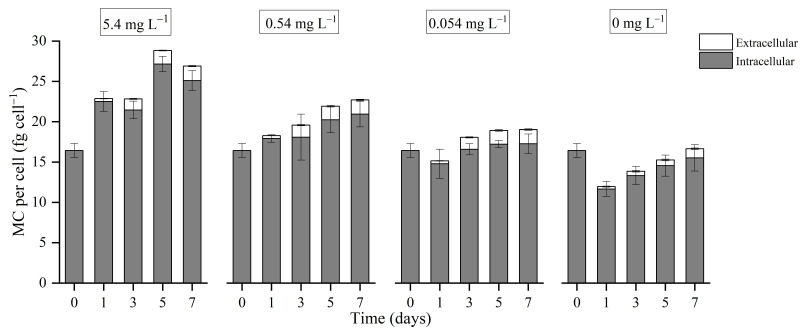
Changes in microcystin (MC) concentration in cultures of *Microcystis aeruginosa* FACHB-905 at different initial phosphorus concentrations. Numbers within the boxes on the top indicate initial phosphorus concentrations in the corresponding groups. Columns with dark grey filling represent intracellular MC. Columns with white filling represent extracellular MC. Error bars indicate standard deviation from three biological replicates.

As shown in Figure 3, intracellular MC accounted for the majority of total MC in *Microcysits aeruginosa* FACHB-905 cells during the course of sampling. After culturing in media with different initial phosphorus concentrations for one day, the total MC concentrations in the 5.4 mg L^−1^, 0.54 mg L^−1^, 0.054 mg L^−1^ and 0 mg L^−1^ cultures changed to 22.86 fg cell^−1^, 18.28 fg cell^−1^, 15.22 fg cell^−1^ and 11.96 fg cell^−1^, respectively. Thereafter, the total MC concentrations in the 5.4 mg L^−1^ and 0.54 mg L^−1^ cultures were consistently higher than that in the 0.054 mg L^−1^ and 0 mg L^−1^ cultures.

### 3.4. Overview of Quantitative Proteomics and Differentially Expressed Proteins

Cells cultured at different initial phosphorus levels for 7 days were harvested for iTRAQ analysis. A relatively long incubation time was adopted in the present study to survey the quantitative proteomic response of *Microcystis* cells to phosphorus limitation acclimation. In total, 313,865 spectra were obtained from the iTRAQ LC-MS/MS analysis, among which 48,717 unique spectra met the stringent confidence identification standard and were further matched to 2567 unique proteins (Table S1), corresponding to approximately 47.14% proteome coverage. According to the threshold mentioned above, 146 and 201 unique proteins were differentially expressed in the 0.054 mg L^−1^ and 0 mg L^−1^ group relative to 5.4 mg L^−1^ group, respectively. The number of downregulated proteins was significantly greater than that of upregulated ones (Table 1). Thirty-five upregulated and 82 downregulated differentially regulated proteins were both identified in the 0.054 mg L^−1^ and 0 mg L^−1^ treatments. In total, 230 differentially abundant proteins (94 upregulated and 136 downregulated) were identified in *Microcystis aeruginosa* FACHB-905 upon phosphorus depletion (Table S2).

### 3.5. KEGG Analysis

**Figure 4:**
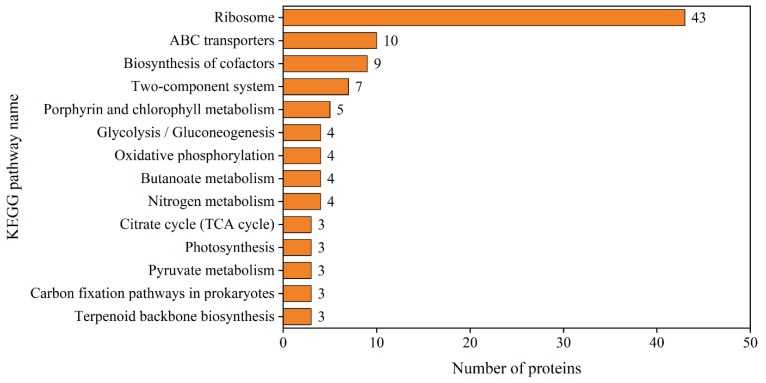
KEGG pathways with at least three differentially abundant proteins in *Microcystis aeruginosa* FACHB-905 upon phosphorus depletion. Y-axis represents KEGG pathway. X-axis numbers represent the number of differentially abundant proteins involved in each KEGG pathway, and the number is also shown next to the bars.

According to KEGG analysis of the 230 differentially abundant proteins, the most enriched KEGG pathway was ribosome (map03010) (Figure 4, with details reported in Table S3), followed by ABC transporters (map02010). Proteins involved in porphyrin and chlorophyll metabolism (map00860), glycolysis (map00010) and nitrogen metabolism (map00910) were significantly differentially expressed upon phosphorus depletion.

### 3.6. Functional Classification

**Figure 5:**
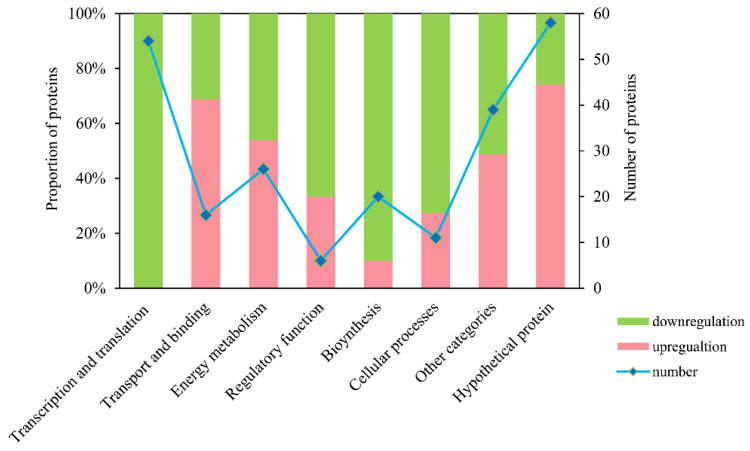
Number and proportion of differentially abundant proteins classified into eight functional categories in *Microcystis aeruginosa* FACHB-905 upon phosphorus depletion. Proteins were assigned into functional categories according to CyanoBase (http://genome.microbedb.jp/cyanobase (accessed on 5 April 2016)) and GO annotation. Proteins with more than a 1.5-fold change (≥1.5 (upregulation) or ≤0.67 (downregulation)) containing at least two unique peptides and a *p*-value below 0.05 were recognized as significantly differentially abundant proteins. Pink columns represent upregulation and green columns represent downregulation. Blue line with diamond symbol represents the number of differentially abundant proteins.

**Figure 6:**
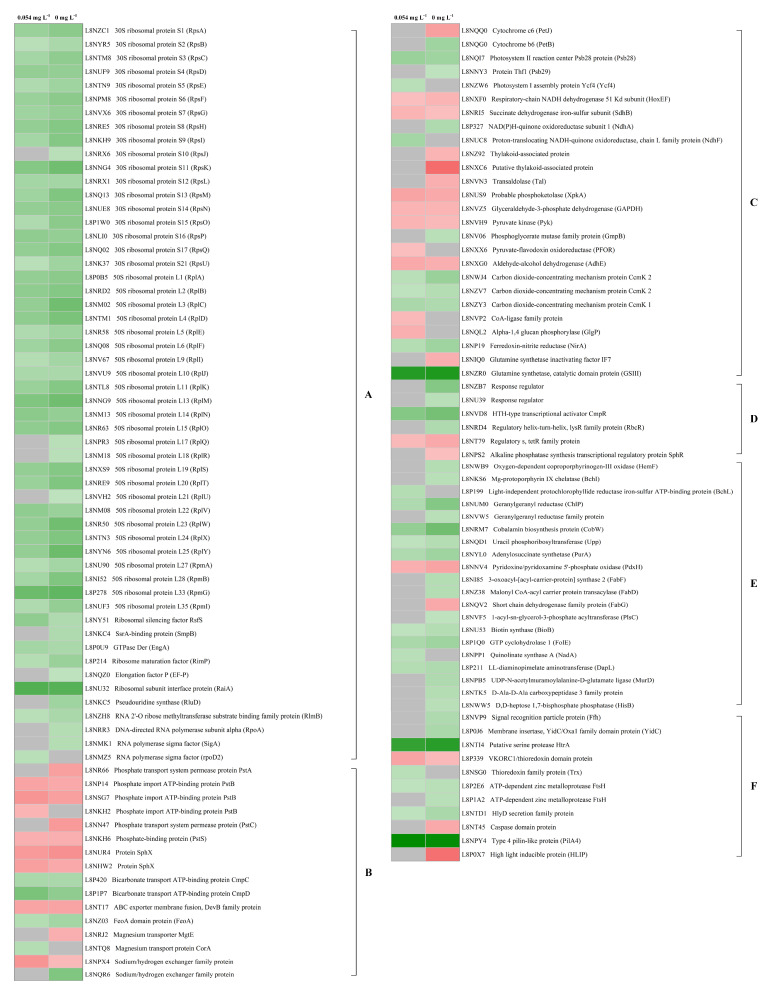

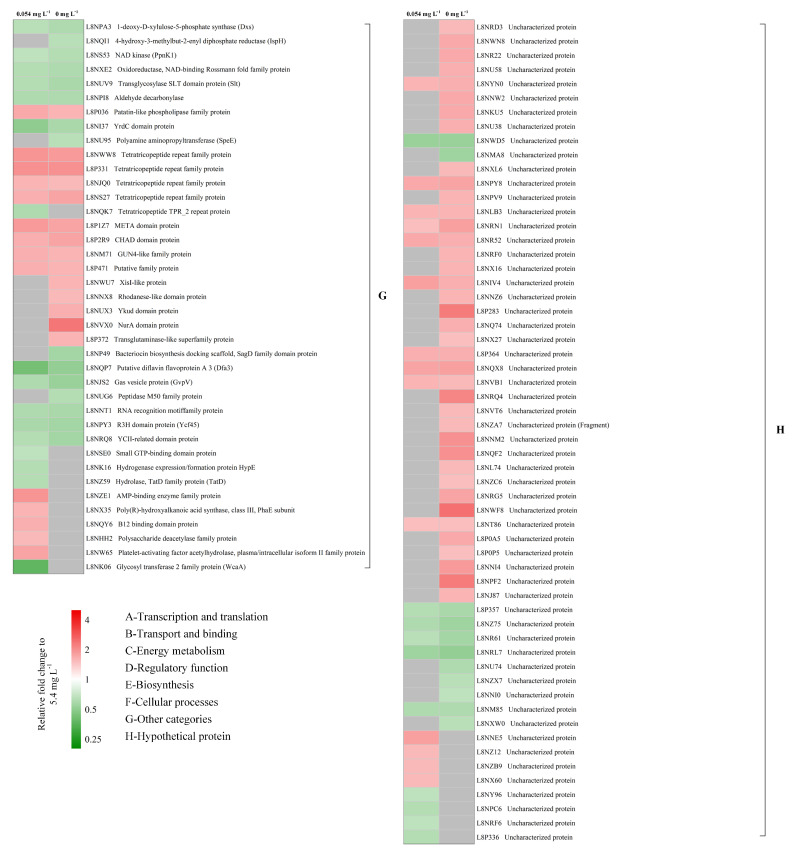
Heat maps of differentially abundant protein changes upon phosphorus depletion arranged into functional categories. Proteins were assigned into functional categories according to CyanoBase (http://genome.microbedb.jp/cyanobase (accessed on 5 April 2016)) and GO annotation. Left column represents the initial phosphorus concentration of 0.054 mg L^−1^ and right column represents the initial phosphorus concentration of 0 mg L^−1^. Colored boxes in each column represent a fold change relative to 5.4 mg L^−1^ phosphorus treatment, red indicates upregulation, green indicates downregulation and grey indicates no significant difference (*p* > 0.05).

The 230 differentially abundant proteins were further manually grouped into eight functional categories according to CyanoBase (http://genome.microbedb.jp/cyanobase (accessed on 5 April 2016), *Microcystis aeruginosa* NIES-843 functional category) [36] and GO annotation (Table S2). A considerable number of differently expressed proteins were categorized to hypothetical proteins (a type of protein whose existence has been predicted but still lacks experimental evidence that it is expressed in vivo; 58 proteins), followed by transcription and translation proteins (54 proteins) (Figure 5). Most of the differentially abundant proteins assigned to transcription and translation, regulatory function, biosynthesis, cellular processes and other categories were downregulated in the phosphorus depletion treatments, while those belong to transport and binding, energy metabolism and hypothetical protein were mostly upregulated. Their relative changes are displayed in heat maps (Figure 6).

The number of differentially abundant proteins within the transcription and translation category is significantly higher than that within other categories, with a majority of 30S (18/21) and 50S (25/35) ribosomal subunits significantly downregulated under phosphorus limitation conditions. Moreover, expression of ribosome maturation protein, RNA-binding proteins and RNA polymerase subunit proteins also decreased.

Proteins within the energy metabolism category are involved in general metabolic pathways, including photosynthesis, respiration, glycogen degradation, glycolysis, the pentose phosphate pathway (PPP), the carbon dioxide-concentrating mechanism (CCM) and the glutamine synthetase (GS)/glutamate synthase (GOGAT) cycle. Under phosphorus limitation conditions, component and assembly proteins related to photosynthesis and respiration were downregulated, while electron transport chain proteins exhibited different changes. Glycogen degradation protein GlgP was significantly upregulated and the same as proteins related to the PPP and glycolysis. Both CCM proteins and most glutamate-glutamine cycle proteins were downregulated, with glutamine synthase (encoded by *glnN*) as one of the most significant proteins.

Proteins within the transport and binding category are related to the transport of ions and molecules across cell membranes. Unsurprisingly, all identified phosphate binding and transport proteins were significantly upregulated. Additionally, the transport and binding of several other important ions, including iron, magnesium and bicarbonate, were remarkably influenced by the depletion of phosphorus.

Proteins within the regulatory function category are involved in the cellular response to stimulus or transcriptional regulation. Our results demonstrate that the regulatory function of *Microcystis* cells was also impacted by phosphorus depletion. Most response regulators were downregulated. Alkaline phosphatase synthesis transcriptional regulatory protein sphR, which is primarily responsible for the transcriptional regulation of genes responding to phosphorus limitation, was significantly upregulated.

Proteins within the biosynthesis category are associated with the biosynthesis of basic substances or important chemicals of cells. Proteins related to the biosynthesis of crucial photosynthetic pigment chlorophyll a; nucleic acid bases purine and pyrimidine; cell envelope components peptidoglycan, lipopolysaccharide and fatty acids; biotin, folic acid and lysine were all significantly downregulated under phosphorus limitation conditions.

Proteins within the cellular processes category are correlated with crucial cellular processes, including protein targeting and translocation, redox regulation, cell division, cell death and chemotaxis. The protein targeting and translocation proteins including signal recognition particle protein and membrane insertase, YidC/Oxa1 family domain protein were both downregulated in the present study. Particularly, phosphorus deficiency dramatically decreased the expression of the chemotaxis protein type 4 pilin-like protein (PilA4), and the PilA4 content in the 0.054 mg L^−1^ and 0 mg L^−1^ treatments was 5-fold less than the 5.4 mg L^−1^ treatment.

## 4. Discussion

In the present study, changes in the protein expression profile and MC production of *Microcystis aeruginosa* FACHB-905 cells grown in both phosphorus depletion and repletion conditions were investigated. Under phosphorus depletion conditions, the growth of *Microcystis* cells was significantly inhibited, as well as the production of MC. The expression levels of proteins related to various metabolic processes changed accordingly.

### 4.1. Phosphorus Acquisition Approach of Strain FACHB-905

The phosphate (Pho) regulon, the general and dominant regulatory mechanism responsible for the bacterial management of phosphorus, is composed of a suite of proteins related to phosphorus transformations and assimilation [37]. Among them, the PstABCS system, as the primary high-affinity and specific inorganic phosphate (P_i_) transport system in bacteria, is the most conserved member of the Pho regulon [38]. In *Escherichia coli* (*E. coli*), these proteins are regulated by PhoR/PhoB, a two-component regulatory system [39]. The two-component system in cyanobacteria is composed of the sensor kinase SphS and the response regulator SphR and works in a similar manner as PhoR/PhoB to sense the P_i_ concentration in the environment and regulate the expression of genes that play a directly role in phosphorus assimilation [40,41]. According to research in *Synechocystis* PCC6803 [42], the high-affinity P_i_-binding protein SphX also belongs to the Pho regulon. As shown in Figure 6, SphX, SphR and proteins within the PstABCS system, were all remarkably upregulated, which could assist in promoting the transport of P_i_. No putative ALP was identified in the present study. Neither any ALP ortholog was found in the predicted proteome of *Microcystis aeruginosa* FACHB-905. The absence of ALP in this strain might be attributed to strain-specific reason, as the ALP-synthesis capacity of *Microcystis aeruginosa* differs significantly at the strain levels [43,44]. We assumed that *Microcystis aeruginosa* FACHB-905 cells did not possess the capability to directly utilize extracellular phosphorylated compounds, and might deal with phosphorus limitation primarily by transporting more available P_i_. The decomposition of phosphorylated compounds outside the cells may mainly be attributed to bacterial ALP activity [45,46]. Our hypothesis was in accordance with the results of Wan et al. [43], which found no extracelluar ALP bound on *Microcystis* cells in both the experimental cultures and the field, but the cells always uptake and store inorganic phosphorus. 

Additionally, Su et al. [47] speculated that SphR, with its well-known function in phosphorus assimilation, was further involved in the transcriptional regulation of genes that play key roles in numerous other biological processes, including carbon and nitrogen metabolism and signal transduction. The function of SphR in regulating essential acid tolerance genes [48] and the clock genes [49] has been discovered. We then hypothesized that phosphorus depletion might trigger the upregulation of SphR, which further regulates genes correlated with essential biological processes promoting survival under phosphorus stressful conditions. This hypothesis may provide clues to the pattern of how cellular biological processes respond to phosphorus availability.

### 4.2. Overall Suppressed Transcription and Translation Processes upon Phosphorus Depletion

Results indicated that the alpha and sigma subunits of RNA polymerase were both significantly downregulated under the present stress conditions. In addition, we discovered that uracil phosphoribosyltransferase (Upp) and adenylosuccite synthetase (PurA), playing a critical role in uridine 5’-monophosphate synthesis and de novo synthesis of adenosine 5′-monophosphate, respectively, were less abundant, which demonstrated that the biosynthesis of (deoxy)nucleotides was inhibited in addition to transcription. The expression of GTP cyclohydrolase 1 and cobalamin biosynthesis protein, which are involved in the biosynthesis of folic acid and cobalamin (both essential cofactor for DNA synthesis) [50], was also remarkably depressed. Overall, these results demonstrated that transcription and related processes were inhibited under phosphorus depletion conditions.

The ribosome is the translation machinery in organisms. Under the phosphorus depletion conditions, the majority of ribosomal proteins, protein GTPase Der, the essential enzyme for the biogenesis of 50S ribosomal subunits [51] and ribosome maturation factor RimP, which has been demonstrated to be crucial for the maturation of 30S ribosomal subunits [52,53], were all downregulated, as previously reported in *Microcystis aeruginosa* PCC7806 under iron-limited conditions [36], in *Prochlorococcus marinus* cells upon nitrogen limitation [54] and in *Arthrospira platensis* suffering heat stress [55]. These results may indicate that the reduction in ribosomes and the subsequent downregulation of protein synthesis are general stress responses. Phosphorus is the primary constituent of RNA, which were the mainly non-storage phosphorus in photosynthetic organisms [56], with ribosomal RNAs (rRNAs) being the single largest investment [57]. Upon phosphorus depletion, available phosphorus tends to be supplied for maintaining basic activities, not synthesizing phosphorus-sink rRNAs. Therefore, the reduction in ribosomal subunits may also be related to fewer rRNAs. In addition, we discovered that translation accuracy could also be affected by phosphorus limitation, as SsrA-binding protein SmpB, ribosomal subunit interface protein RaiA and elongation factor P (EF-P) were all downregulated. These proteins are critical for *trans*-translation [58], anti-miscoding activity [59] and the synthesis of proteins possessing consecutive proline residues [60].

In summary, the inhibition of transcription and translation reflected the suppression of cell growth, in accordance with the significantly lower cell concentration upon phosphorus limitation.

### 4.3. Cellular Changes in Carbon and Nitrogen Metabolism upon Phosphorus Depletion

Glycogen is the main storage mechanism of photosynthetically fixed carbon in cyanobacterial cells. Upon phosphorus depletion, glycogen phosphorylation protein GlgP was significantly more abundant. GlgP catalyzes glycogen degradation and generates d-glucose-1-phosphate in the preparatory, rate-limiting step, the resulting product subsequently participates in glycolysis and the PPP processes [41], the related enzymes of which were upregulated as well. Transaldolase (Tal) catalyzes the 6th step of the PPP, and its increase might indicate the enhancement of PPP activity. In addition, the glycolytic enzymes glyceraldehyde-3-phosphate dehydrogenase (GAPDH) and pyruvate kinase (Pyk) were both significantly upregulated. The increase in GAPDH was congruent with the change in *Synechococcus* sp. WH8102 [61] and WH7803 [41] under similar conditions. Pyruvate, the end product of glycolysis, is chemically active and can be processed into numerous molecules. Under dark conditions, pyruvate could be oxidized to acetyl-coenzyme A (acetyl-CoA) and CO_2_ by pyruvate-flavodoxin oxidoreductase (PFOR). Acetyl-CoA could further be transformed to ethanol by AdhE. Both PFOR and AdhE were significantly upregulated in the present study. To sum up, the above results indicated a significant increase in carbon catabolism of *Microcystis* cells suffering phosphorus depletion.

One of the most significantly downregulated proteins in our study was GSIII (nearly four-fold down-regulated upon phosphorus depletion). GS is the key point of ammonium assimilation in cyanobacteria, which catalyzes the condensation reaction between glutamate and ammonium to produce glutamine [62]. There are two types of GS, GSI and GSIII, encoded by *gln*A and *gln*N, respectively, that are governed by different transcriptional and posttranscriptional regulations [63]. Even though GSI abundance was not dramatically affected under the phosphorus stress conditions, its inactivating factor IF7 was remarkably upregulated. IF7 inactivates GSI by protein-protein interaction [64]. Its increase may suggest that less bioactive GSI could participate in nitrogen assimilation. All these results indicated that nitrogen assimilation was suppressed under phosphorus depletion conditions. Together with the increase in carbon catabolism, these results show specific meaning for the intracellular balance among carbon, nitrogen and phosphorus of *Microcystis* cells under phosphorus depletion conditions.

### 4.4. Effects of Phosphorus Depletion on Photosynthesis

Regarding the structure of the photosynthetic apparatus and photosynthetic electron transport chain (PETC), only proteins functioning in assembly or stabilization of photosystems were found to be downregulated, including photosystem II (PSII) reaction center Psb28 protein, which is likely related to the biogenesis of the PSII inner antenna CP47 [65], Psb29 (Thf1), which interacts with photosystem I (PSI) to stabilize the PSI complex [66], and photosystem I assembly protein Ycf4, which discovered not essential for PSI assembly [67]. Primary protein subunits of PSII and PSI, particularly D1 (PsbA), D2 (PsbD), PsaA and PsaB, were not differentially abundant among different treatments, which might indicate that even suffering a relatively long time of phosphorus depletion, the PS structure of *Microcystis* cells remained complete. This characteristic could be beneficial for rapidly recovery and regrowth of this organism once receiving phosphorus supplement [44].

The down-regulated activity of photosynthesis upon phosphorus depletion [44,68] could be due to inhibited electron transfer and remarkably reduced photosynthetic pigment. In the present study, protein cytochrome b6 PetB, the subunit of the cytochrome *b*_6_*f* (Cyt *b*_6_*f*) complex, was significantly less abundant. The Cyt *b*_6_*f* complex mediates the photoinduced electron transfer between PSII and PSI and promotes protons pumped across the membrane into the thylakoid lumen [69]. Its decrease may result in fewer electrons for PETC and the reduction in the proton gradient for ATP synthesis [70].

Chlorophyll a is the most important photosynthetic pigment in cyanobacteria and is crucial for light harvesting and energy conversion. We discovered that under phosphorus depletion conditions, proteins involved in the biosynthesis of chlorophyll a were significantly less abundant, including oxygen-dependent coproporphyrinogen-III oxidase HemF, Mg-protoporphyrin IX chelatase BchI and light-independent protochlorophyllide reductase iron-sulfur ATP-binding protein BchL, which catalyze the 8th, 10th and 16th steps of chlorophyll a synthesis from glutamic acid, respectively [71], as well as geranylgeranyl reductase ChlP, which participates in the final steps of chlorophyll a synthesis [72]. Presumably it was the downregulation of these proteins that led to a drop in the chlorophyll a content, which indeed was in accordance with the experimental results. After culturing at different phosphorus concentrations for 7 days, the chlorophyll a concentration per cell decreased with lower phosphorus concentration, and the decrease was more significant in the 0.054 mg L^−1^ and 0 mg L^−1^ groups (*p* < 0.01), as has been discovered in previous research [44,68]. The decline in the biosynthesis of chlorophyll a and its content might also reflect the acclimation mechanism of avoiding excessive light energy of *Microcystis* cells under phosphorus depletion conditions, as a type of photoprotection method [73]. The increase in the ratio of carotenoid/chlorophyll and induction of high light inducible protein HLIP may also assist in photoprotection [74,75].

Furthermore, as the final electron’s deposition pool, the expression of proteins related to carbon assimilation were also changed. CO_2_ concentrating mechanism (CCM)-related proteins, including bicarbonate transport ATP-binding protein CmpC, CmpD and HTH-type transcriptional activator CmpR, were all less abundant. Cmp proteins participate in bicarbonate uptake and facilitate the transfer of bicarbonate across the cell membrane [76]. Moreover, the major shell proteins of carboxysomes, CcmK1 and CcmK2, were also less abundant than the 5.4 mg L^−1^ group. The carboxysome is the place where bicarbonate is converted to CO_2_ and assimilated by ribulose 1,5-bisphosphate carboxylase/oxygenase (RuBisCO). CcmK1 and CcmK2 assemble into flat facets of the polyhedral shell of carboxysomes in their hexameric form [77,78]. Their downregulation may imply fewer carboxysomes for carbon fixation. However, in our study, it seems that RuBisCO large and small subunit proteins are not significantly affected by phosphorus deprivation, which requires further investigation.

### 4.5. Phosphorus Deprivation Indirectly Affects the Production of MC

All MC synthetic proteins were identified in our study, and their identification and quantitation information are listed in Table 2. Relative fold changes listed in Table 2 were statistically significant (*p* < 0.05). Based on the stated threshold of a 1.5-fold change, the expression of any MC synthetase was not significantly changed. However, according to Figure 3, the MC cell quota of FACHB-905 cells decreased dramatically with less available phosphorus in the medium. The inconsistency could also be found between *mcy* gene transcript and MCs production as reported in other literatures [79,80,81]. It seems that phosphorus depletion does not directly regulate the production of MC on the genetic level, but on the cellular level. The constant expression of MC synthetase guaranteed the potential toxicity of *Microcystis* cells, as quite variously biological function that MC possesses [82], while the hard phosphorus depletion conditions could not support the relevant synthesis of MC.

Firstly, less raw materials for MC synthesis resulted from phosphorus deprivation might be one reason. MC is chemical rich in carbon and nitrogen (49 carbon atoms and 10 nitrogen atoms in each molecule) which accounted for approximately 0.3% dry weight per cell of FACHB-905 cells. Furthermore, its structure contains several complex components including Adda ((2*S*,3*S*,8*S*,9*S*)-3-amino-9-methoxy-2,6,8-trimethyl-10-phenyldeca-4,6-dienoic acid) and Mdha (N-methyldehydroalanine) [83]. As discussed above, carbon and nitrogen assimilation and other primary cellular processes were all inhibited under phosphorus depletion conditions, indicating a slowing down of cellular metabolism, thus less intracellular carbon, nitrogen and relative unique amino acids could be used for MC synthesis. Secondly, energy limitation might also inhibit MC production. It has been widely discovered that MC synthesis is an energy consumption process and positively correlated with the energy state of producers [12,81,84]. Furthermore the energy for MC biosynthesis was mostly contributed by ATP derived from photosynthesis activities [21]. The tight relationship between photosynthesis and MC synthesis has been validated by several studies [21,81,85]. ATP shortage might occur due to the significantly suppressed photosynthetic activity under phosphorus deficiency conditions, leading to less MC production. What is more, as MC content could be controlled at the level of transcription, translation and enzyme activities [30], it may be the enzyme activities of MC synthetic proteins that were inhibited by phosphorus depletion, not the abundance of MC synthetic proteins, leading to decreased MC production.

## 5. Conclusions

The results of this study revealed that orthophosphate transport system proteins played a critical role in the surviving strategies of *Microcystis aeruginosa* FACHB-905 cells facing phosphorus depletion, which has no intrinsic ability to produce alkaline phosphatase. Phosphorus depletion remarkably inhibited the primary metabolic processes of *Microcystis* cells, including transcription, translation and carbon and nitrogen assimilation, while induced the process of carbon catabolism, with structures of photosystems remaining intact. The expression of MC synthetase was not significantly changed upon phosphorus deprivation, while MC content was significantly suppressed. We supposed that phosphorus depletion indirectly regulated the production of MC by the inhibition of relative metabolic processes and energy production.

## Figures and Tables

**Table 1:** Number of differentially abundant proteins in *Microcystis aeruginosa* FACHB-905 upon phosphorus depletion. The differentially abundant proteins reported were upregulated or downregulated in the phosphorus depletion treatments (0.054 mg L^−1^ and 0 mg L^−1^ initial phosphorus concentrations) with a fold change relative to 5.4 mg L^−1^ phosphorus treatment. Proteins with more than a 1.5-fold change (≥1.5 (upregulation) or ≤0.67 (downregulation)) containing at least two unique peptides and a *p*-value below 0.05 were recognized as significantly differentially abundant proteins.

Relative to 5.4 mg L^−1^ Phosphorus Treatment	0.054 mg L^−1^	0 mg L^−1^	0.054 mg L^−1^ and 0 mg L^−1^
Upregulation	48	81	94
Downregulation	98	120	136
Total	146	201	230

**Table 2:** Relative abundance of ten microcystin synthetic proteins in *Microcystis aeruginosa* FACHB-905 grown upon phosphorus depletion. All relative abundance listed were statistically significant (*p* < 0.05).

Accession Number ^a^	PROTEIN NAME	Gene ^b^	Unique Peptides ^c^	0.054 mg L^−1^ vs. 5.4 mg L^−1^	0 mg L^−1^ vs. 5.4 mg L^−1^
L8NWM7	Nonribosomal peptide sythetase McyA protein	*mcyA*	9	0.79	0.79
L8NXN8	Nonribosomal peptide synthetase McyB protein	*mcyB*	20	1.15	–
L8NXL3	Nonribosomal peptide synthetase McyC protein	*mcyC*	12	–	–
L8NTG4	Polyketide synthase type 1 McyD	*mcyD*	8	0.84	0.75
L8P064	Polyketide synthase peptide synthetase fusion protein McyE	*mcyE*	15	–	0.75
L8NXL9	Asp/Glu racemase McyF	*mcyF*	8	–	–
L8NXP2	Peptide synthetase polyketide synthase fusion protein McyG	*mcyG*	13	–	0.87
L8NWN2	ABC transporter ATP-binding protein McyH	*mcyH*	1	0.76	0.71
L8NTH0	Dehydrogenase McyI	*mcyI*	8	–	0.95
L8P068	O-methyl transferase McyJ	*mcyJ*	8	0.86	0.78

^a^ Accession number from the UniProt database; ^b^ Gene encoding the corresponding protein; ^c^ Unique peptides for protein identification and quantitation; – indicates protein quantitation not statistically significant according to Student’s *t* test (*p* > 0.05).

## Data Availability

Not applicable.

## Supplementary Materials

The following are available online at https://www.mdpi.com/article/10.3390/microorganisms9061183/s1, Table S1. Characteristics of the 2567 identified proteins in *Microcystis aeruginosa* FACHB-905. Table S2. Differentially abundant proteins in *Microcystis aeruginosa* FACHB-905 upon different phosphorus concentrations treatment. Values highlighted in red represent upregulation while those in green represent downregulation of the protein relative to the 5.4 mg L^−1^ treatment. Table S3. KEGG pathway enrichment analysis of differentially abundant proteins in *Microcystis aeruginosa* FACHB-905 upon phosphorus depletion. Only pathways with at least three differentially abundant proteins were listed.
